# Emerging evidence of a link between the polycystins and the mTOR pathways

**DOI:** 10.1186/1755-8417-2-6

**Published:** 2009-10-28

**Authors:** Alessandra Boletta

**Affiliations:** 1 Dulbecco Telethon Institute (DTI) at Dibit, San Raffaele Scientific Institute, Via Olgettina 58, 20132 Milan, Italy

## Abstract

Autosomal dominant polycystic kidney disease (ADPKD) is a genetic disease characterized by the formation of renal cysts. This disease can be caused by mutations in two genes, *PKD1 *and *PKD2*, which encode polycystin-1 (PC-1) and -2 (PC-2), respectively.

PC-1 is a large plasma membrane receptor involved in the regulation of several biological functions and signaling pathways, and PC-2 is a calcium channel of the TRP family. The two proteins associate in a complex to prevent cyst formation, but the precise mechanism(s) involved remain largely unknown.

This review will focus on recent advances in our understanding of the functions of polycystins and their role in signal transduction.

Increased activity of the mammalian target of rapamycin (mTOR) kinase has been observed in cysts found in ADPKD tissues. Rapamycin has been shown to have beneficial effects in rodent models of polycystic kidney disease, prompting the initiation of pilot clinical trials with human patients. Furthermore, a direct role for PC-1 in the regulation of cell growth (size) via mTOR has recently been demonstrated.

Major advancements in the study of mTOR biology have highlighted that this kinase exists in association with two different complexes, mTOR complex 1 (mTORC1) and mTOR complex 2 (mTORC2). The mTORC1 complex regulates cell growth (size), proliferation, translation and autophagy, and mTORC2 regulates the actin cytoskeleton and apoptosis. Interestingly, mTORC2 has been shown to contain the kinase responsible for the phosphorylation of Akt at Serine 473. Previous studies have shown that PC-1 controls the PI 3-kinase/Akt cascade to regulate apoptosis and the actin cytoskeleton, suggesting that this receptor might regulate mTOR at several levels.

This review aims to discuss three different, inter-related themes emerging from the literature: (i) studies performed in our and other laboratories collectively suggest that PC-1 might be able to differentially regulate the two mTOR complexes; (ii) several studies point to genetic and functional cross-talk between the PKD and TSC genes, although the molecular details remain obscure; and (iii) studies performed in mammals and in the unicellular algae *Chlamidomonas Reinhardtii *might highlight a link between cilia, regulation of cell size and regulation of the cell cycle.

## ADPKD: genetics and proposed mechanisms of cystogenesis

Autosomal dominant polycystic kidney disease (ADPKD) is a frequent genetic disease, affecting between 1:500 and 1:1000 of the general population [1-3]. The hallmark of the disease is bilateral renal cyst formation, although a plethora of extra-renal manifestations have been reported. These include liver and pancreatic cysts as well as cardiovascular complications (for review of the clinical aspects of the disease, see [1]). Two genes have been linked to this disease: *PKD1*, which is mutated in 85% of cases, and *PKD2*, which is mutated in most of the remaining cases. Numerous mutations have been reported along the length of these two genes; most are loss-of-function mutations predicted to inactivate the affected allele [1-3].

### Genetic mechanism of cystogenesis

ADPKD is inherited in an autosomal dominant manner, but it has been suggested that it is recessive at the molecular level [4]. Analysis of the epithelia derived from renal or liver cysts has revealed that the normal inherited allele undergoes a somatic mutation, resulting in homozygous loss of either the *PKD1 *or *PKD2 *gene [4,5]. This 'second hit' causes a single affected epithelial cell to over-grow and generate a clonal outpouching diverticulum, which eventually disconnects from the renal tubule. In some of the cysts in which homozygous inactivation of one *PKD *gene (either 1 or 2) is not observed, mutations in the other *PKD *gene have been found, suggesting that trans-heterozygosity of the two genes is sufficient to induce cyst formation [6]. Based on these findings, a 'threshold model' of cystogenesis has been proposed, in which heterozygosity is not sufficient to induce renal cystogenesis, but a minimum level of activity of the *PKD1 *and *PKD2 *gene products, the polycystins, is required to prevent cystogenesis. Any dysfunction causing polycystin activity to fall below such a threshold would lead to cyst formation, even though a complete loss of function does not appear to be necessary. Several findings from studies in animal models appear to support this model: (i) heterozygosity of the *Pkd1 *or *Pkd2 *gene is not sufficient to cause renal cyst formation in mice, possibly due to a low rate of second hits in the murine kidney, whereas homozygous inactivation causes renal cystogenesis *in utero *[7,8]; (ii) conditional inactivation of the second allele in the kidney results in renal cystogenesis with a variably severe phenotype depending on the time of inactivation [9,10]; (iii) a mouse model carrying an unstable *Pkd2 *allele that spontaneously undergoes somatic inactivation displays the ADPKD phenotype when crossed with a *Pkd2*-null mouse [11]; (iv) trans-heterozygous *Pkd1*^+/-^:*Pkd2*^+/- ^mice display markedly more severe cystic kidney disease than single heterozygous mutants [12]; and (v) a mouse model carrying an aberrantly spliced variant of the *Pkd1 *gene, lowering its expression to 13-20% of normal levels, suffers renal cystogenesis [13].

Intriguingly, a recent study has identified ADPKD families carrying homozygous inheritance of mild mutations in the *PKD1 *gene, which resulted in PKD; this finding further suggests that gene dosage might be important [14]. Thus, loss of function (complete or partial) of the *PKD1 *and *2 *genes appears to be the most common mechanism of renal cystogenesis in ADPKD.

It should be taken into account, however, that overexpression of the human or murine *PKD1 *or *2 *genes in mice is sufficient to induce renal cyst formation [15-17]. Therefore, we cannot exclude the possibility that a few cysts in ADPKD might result from enhanced activity of the two genes.

Finally, although haplo insufficiency does not seem to be involved in the mechanism of cystogenesis, it is most likely the cause of some of the other systemic and non-focal manifestations of the disease, such as some of the cardiovascular defects. Evidence from mice and cellular models seem to support this idea [18,19].

### Molecular mechanism(s) of cystogenesis

The studies mentioned above strongly point to loss of function as the most common mechanism of cyst formation, but how does a lack of PKD gene products result in cystogenesis? A large body of literature has been published on the characterization of the cystic epithelia of ADPKD kidneys. More recently, the generation of mouse models mimicking the disease has allowed for the confirmation of several of the original observations made in humans. These studies have helped to build a general consensus on some key features of these epithelia: (i) matrix deposition appears to be defective, with thickening of the basement membrane [20]; (ii) increased death by apoptosis has been consistently observed in human and in mouse specimens [10,21,22]; (iii) proliferation seems to be enhanced in the cystic epithelia, although to a low extent, causing a very slow expansion of the cystic epithelia over time [23] and making it difficult to visualize sharp differences in proliferation markers in slowly progressing renal cystic models that mimic the human disease [10]; and (iv) there is enhanced fluid secretion towards the lumen of the cyst, mostly driven by cAMP [24,25].

More recently, upregulation of the mTOR pathway has been observed in the cystic kidneys of ADPKD specimens, as well as in a series of rodent models of PKD [[26-29], also see below]. Rapamycin, an inhibitor of mTOR, has been shown to protect these animal models, as well as ADPKD patients, from cyst expansion, possibly by reducing proliferation [26,29-31].

Currently, drugs targeting proliferation or cAMP production (and fluid secretion) are considered the most promising approaches to slow down cyst expansion and some are currently being tested in clinical trials [2,3].

Although enhanced proliferation and secretion might partly explain the mechanism involved in the expansion of the cysts, these factors do not explain how a cyst initially arises. A very appealing recent hypothesis has been raised that states that the planar cell polarity (PCP) of the renal tubule might be affected in cystic kidney disease and could be the initiating defect [32]. Several lines of evidence support this hypothesis.

First, it has been shown that the distal and collecting duct tubules undergo a PCP program of oriented cell division, leading to their elongation in the postnatal kidney. This programme appears to be disrupted in mice developing cysts in the collecting duct [33,34].

Second, a different mechanism, which still relies on PCP, has been shown to be defective during embryonic development: convergent extension movements might contribute to tubule elongation in developing nephrons [35]. This process appears to be defective in a mouse model of cystic kidney disease due to inactivation of Wnt9b [35].

Third, the inversin gene, which is mutated in nephronophthisis, another cystic kidney disease, has been shown to regulate the Wnt pathway and to allow for a switch between canonical (β-catenin dependent) and non-canonical (also called PCP) pathways in response to the bending of cilia [36].

Finally, a recent study has shown that inactivation of one of the mammalian orthologs (Fat4) of the tumour-suppressing atypical cadherin Fat, a major regulator of PCP in *Drosophila melanogaster*, in the kidney results in cystic kidney disease and defective mitotic spindle orientation [37].

Taken together, these data suggest that cytoarchitectural defects caused by dysfunctional PCP might be the basis of cyst initiation. It should be noted, however, that no evidence has been provided to date to show that the *PKD1 *or *2 *genes are involved in the regulation of PCP. Bonnet *et al*. have recently reported defective mitotic spindle orientation in *Pkd1*^+/- ^kidneys. However, this defect was accompanied by the presence of only a few cysts, possibly due to a low rate of somatic inactivation of the *Pkd1 *gene in the murine kidney as mentioned above [28]. Defective mitotic spindle orientation in the absence (or in the presence of a very low rate) of renal cyst formation suggests that this defect might not be sufficient to cause renal cystogenesis. The same authors also showed that haplo insufficiency of the *Pkd1 *gene does not cause PCP defects in the inner ear [28]. This result is not surprising when considering that most of the phenotypes caused by mutation of the *Pkd1 *or *Pkd2 *genes cannot be observed in heterozygotes. In order to determine the potential role of these two genes in PCP, studies should be performed on homozygously mutant embryos or by tissue-specific inactivation of both alleles of the *Pkd1 *or *Pkd2 *genes in the cochlea and/or in the kidney.

## The polycystins

The gene products of *PKD1 *and *2*, polycystin-1 and -2 (PC-1 and PC-2), assemble through coiled-coil domains present in their intracellular C-termini to form a functional complex, the activity of which is believed to be essential to prevent renal cystogenesis [38-41]. Most of their activities have been attributed to this PC-1/2 complex, which explains the identical phenotype observed in ADPKD1 and ADPKD2 patients [41]. However, these two proteins are also expected to have independent functions and it has been shown that the establishment of left-right asymmetry in the developing mouse embryo requires PC-2, but not PC-1 [42].

### The biochemistry of the polycystins

**Figure 1:**
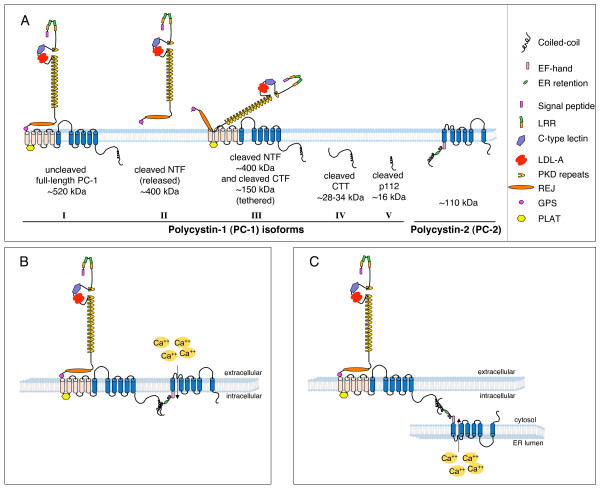
**Schematic representation of the polycystins**. **A**. Polycystin-1 is a large plasma membrane receptor that undergoes a series of cleavage events to generate several different species co-existing within the same cell and most likely carrying out distinct functions. The protein exists as an uncleaved polypeptide of 4302 amino acids (aa) (**I**) and can be cleaved at its G-protein coupled proteolytic site, generating an N-terminal fragment (NTF) that can be released (**II**) or remain tethered to the C-terminal fragment (CTF) (**III**) [48]. Two additional products generated by cleavage at yet-to-be-identified sites release either the entire C-terminal tail (**IV**) [52] or the last 112 aa (**V**) [54]. **B and C**. PC-1 and PC-2 have been shown to interact through coiled-coil domains located in their cytoplasmic C-terminal tail. The precise localization and topology of the complex remains to be determined. The two proteins might co-localize at the plasma membrane, where PC-2 would regulate calcium influx from the extracellular compartment (**A**) [39]. This might occur in some subcellular compartments such as the primary cilium. Alternatively, the plasma membrane pool of PC-1 might interact with the endoplasmic reticulum (ER) pool of PC-2, regulating its calcium release from the ER (**B**) [60].

PC-1 is a large plasma membrane receptor consisting of 4302 amino acids (aa), with an extracellular N-terminal portion of ~3,000 aa, 11 transmembrane domains and a relatively short intracellular C-tail of 198 aa [43-45]. The N-terminus contains a novel combination of protein-protein interacting domains, including leucine-rich repeats (LRRs), a C-type lectin domain, 16 PKD repeats (IgG-like domains) and an REJ domain, which is named for its homology with the sea urchin Receptor for Egg Jelly (Figure 1A). The protein is predicted to have a molecular mass of 462 kDa [43,44].

The study of PC-1 biochemistry and function has been challenging due to its low abundance and large size. Studies using heterologously over-expressed, human, full-length PC-1 have shown that the protein is heavily glycosylated, reaching a final estimated mass of ~520 kDa [46,47]. Just N-terminal to the first transmembrane domain, the protein contains a G-protein coupled receptor proteolytic site (GPS), an auto-proteolytic site that results in the cleavage of PC-1 into two fragments: an N-terminal fragment (NTF, ~400 kDa) corresponding to the extracellular portion of the protein and a C-terminal fragment (CTF, ~150 kDa) composed of the remainder of the protein (Figure 1A) [48,49]. The cleaved NTF can either be released or remain tethered to the CTF fragment. PC-1 is not completely cleaved at the GPS and the full-length uncleaved protein co-exists with the cleaved version in cells (Figure 1A) [48]. Cleavage at the GPS has been shown to be essential for PC-1 function both *in vitro *and *in vivo *[48,50]. Mice carrying a single point mutation at the GPS, which prevents the cleavage of PC-1, survive to birth, but develop polycystic kidney disease, resulting in renal failure in the first few weeks of life [50]. Therefore, it appears that the GPS is essential for some of the functions of PC-1, including preventing renal cyst formation in the distal and collecting duct tubule; the uncleaved product is also likely to play an important role [50].

The intracellular C-tail of PC-1 contains a coiled-coil domain that is responsible for mediating interaction with PC-2 and other proteins [38-40] and a consensus site for interaction with heterotrimeric G-proteins [51]. The C-tail has also been shown to be cleaved at a minimum of two different sites, generating two distinct products (Figure 1A): one 28 to 34-kDa product containing the entire intracellular C-tail of PC-1 (CTT) [52,53] and a second ~16-kDa product (p112) [54]. Both of these products have been observed to interact with transcription factors (β-catenin and STAT6, respectively) and to translocate into the nucleus [53,54]. The extent of the cleavage of CTT and its transcriptional activity depend on PC-2 and the regulation of intracellular calcium stores [55]. Identification of the cleavage sites generating these products, as well as identification of the endogenous fragments, has not yet been achieved.

Finally, the intracellular C-tail contains putative phosphorylation sites, some of which have been shown to be phosphorylated using *in vitro *kinase assays [56,57].

Polycystin-2 (PC-2, also called TRPP2) is a 968-aa membrane protein containing six transmembrane domains, with both N- and C-terminal tails facing the cytoplasm (Figure 1A) [58,59]. PC-2 shares homology with the transient receptor potential (TRP) family of calcium channels and, indeed, it has been shown to act as a calcium channel in either the plasma membrane or the endoplasmic reticulum [39,60]. PC-2 contains a ciliary targeting domain in its N-terminal tail [61] and both an EF-hand and a coiled-coil motif in its C-terminal portion [40,59]. Biochemical analysis has revealed that PC-2 is a 110-kDa glycosylated membrane protein that appears to be mostly localized to the ER compartment, as documented by its complete sensitivity to endoH [62]. A minimal amount of the protein might reach the plasma membrane under physiological conditions [61-64]. The amount of protein that reaches the plasma membrane might be greatly enhanced by over-expression of PC-1, which has also been suggested to be essential for PC-2 trafficking to the plasma membrane, where the two proteins might form a functional complex that regulates calcium influx (Figure 1B) [39,63]. An alternative model that takes into account the ER localization of the PC-2 channel and its concomitant interaction with PC-1, which is localized at the plasma membrane, is illustrated in Figure 1C. In this scheme, the calcium channel activity of PC-2 would still be regulated by PC-1, but it would mostly cause ER calcium release rather than calcium influx from the extracellular compartment. Recent studies have proposed that both mechanisms might take place within the same cell [65].

Post-translational modifications of PC-2 include phosphorylation by serine/threonine kinases (casein kinase II, GSK3β), which influences its subcellular localization by regulating its association with other molecules [65,66].

### Localization and function of the polycystins

Given the complexity of the processing of the polycystin-1 and -2 complex and the coexistence of different isoforms within cells, it is not surprising that the subcellular localization and functional characterization of these proteins has uncovered a very complicated picture. However, it should also be considered that detection of endogenous polycystin-1 has been problematic due to the low specificity of the antibodies directed against the protein and/or to the very low abundance of this receptor.

The plasma membrane pool of the PC-1/2 complex has been localized to cell-cell junctions [41,46,67-69], where PC-1 has been shown to regulate the rate of adherens junction formation [68,69] and the mechanical force of cell-cell adhesion [70]. PC-1 has also been localized to cell-matrix interacting plaques [71]. Finally, both polycystins have been localized to primary cilia [72,73]. These are long, thin, microtubule-based, non-motile structures that protrude from many different cell types. In epithelial and endothelial cells that develop an apico-basal polarity, cilia appear on the apical side, where they are believed to be essential mechanosensors responding to flow [74]. Increasing evidence suggests that, in renal epithelial cells and endothelial cells, both polycystins localize to cilia, where PC-1 acts as a mechanosensor [75,76], possibly by virtue of its long N-terminal domain. This domain is able to stretch in response to mechanical forces [77,78] and, in doing so, might activate PC-2, which opens its channel pore and allows calcium to enter the cell [75,76].

In addition to its intracellular distribution, the PC1/2 complex has recently been identified in urinary exosomes, small double-membrane vesicles released from the apical side of epithelial cells that are believed to allow for communication between distant cells [79]. These exosomes have also been shown to associate and fuse to primary cilia in bile ducts, raising the possibility that they might enable communication between distant epithelia within a tubule through exosome-cilia interactions [79].

PC-1 and PC-2 have also been implicated in several potential biological functions. Both have been shown to protect cells from apoptosis under different stress conditions [80-82]. In addition, the PC-1/2 complex has been found to inhibit cell proliferation through the activation of the JAK2/STAT1/p21 signaling pathway [83], further enhanced by a PC-2-Id2 interaction [84]. These two proteins have also been demonstrated to be important regulators of cell migration and epithelial morphogenesis using *in vitro *three-dimensional models [70,80,85,86]. Recently, studies conducted on each protein separately have suggested that they regulate the protein translation machinery to some extent; PC-1 has been shown to regulate mTOR downstream effectors that are known to act on translation, such as S6K1 and S6 ribosomal protein and the 4EBP1/eIF4E complex [87], and PC-2 has been shown to regulate another translation initiation factor, eIF2alpha, by regulating its phosphorylation by PERK in response to ER stress [88]. Interestingly, microarray studies performed on ADPKD tissues have revealed that several genes upregulated in cystic kidneys belong to the translation machinery [89]. Increased translation in ADPKD might explain the observed cellular hypertrophy manifestations (see below) and could contribute to deregulated proliferation [23].

Finally, PC-1 has been shown to control several additional signaling cascades in line with its role as a receptor; the Wnt cascade [53,90], AP-1 [91], PI3kinase/Akt [70,81], GSK3β [70], STAT6 [54], the calcineurin/NFAT [92] pathway and the ERK and mTOR cascades [87] have all been reported to be regulated by PC-1. This review will focus on the regulation of mTOR and its complexes by PC-1.

## The mTOR pathway, the mTOR complexes and the feedback loops

mTOR (mammalian target of rapamycin) is a well-conserved serine/threonine kinase of the PIKK family (phosphoinositide 3-kinase-related kinase) that is involved in several processes, including cell growth regulation, proliferation, regulation of the cellular cytoskeleton and cell survival [93-95].

**Figure 2:**
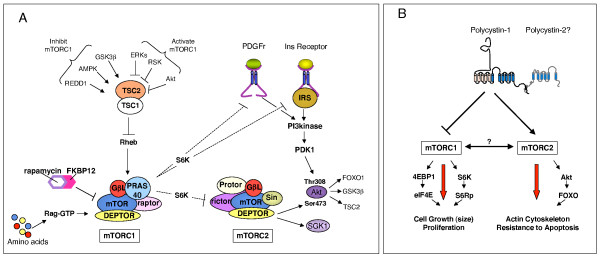
**Overview of the two mammalian targets of rapamycin (mTOR) complexes and their potential regulation by the polycystins**. **A**. Schematic overview of the composition of mTOR complex 1 (mTORC1) and mTOR complex 2 (mTORC2) and cross-talk between them. mTORC1 contains mTOR, raptor, GβL/mLST, PRAS40 and DEP domains interactor of mTOR (DEPTOR). It can be regulated by a variety of activating or inhibitory cascades, as well as by amino acids capable of associating with Rag-GTP, leading to its association with mTORC1 to enhance its activity. One of the effectors of mTORC1, S6K1/2, regulates a negative feed-back loop at several levels. It is able to regulate insulin signalling by phosphorylating and inducing the degradation of IRS [111-113], and PDGF signalling by regulation of PDGF receptor levels [110]. In addition, S6K1/2 can phosphorylate rictor [114]. mTORC2 contains mTOR, Rictor, GβL/mLST, mSin and Protor. mTORC2 can phosphorylate Akt at Serine 473, regulating its specificity towards different substrates. Akt, in turn, can phosphorylate Tuberin (TSC2), potentially placing mTORC1 downstream of mTORC2 (see text). mTORC2 can also phosphorylate SGK1. **B**. Schematic representation of the effect of PC-1 on the two mTOR complexes. PC-1 has been described to inhibit the mTORC1 complex [87] whereas mTORC2 seems to be upregulated, since Akt phosphorylation at Serine 473 is enhanced by overexpression of PC-1 [70,81]. The role of PC-2 in the regulation of these cascades and their precise mechanism of regulation remain to be clarified.

mTOR assembles into two distinct complexes whose molecular components have been partly characterized: complex-1 (mTORC1) and complex-2 (mTORC2) [95]. Besides mTOR itself, the two complexes contain common molecules, such as GβL/mLST8 and DEPTOR [96], although they differ in their other components; mTORC1 contains Raptor [97] and PRAS40 [98], whereas mTORC2 contains Rictor [99], mSin1 [100,101] and Protor [102] (Figure 2A).

mTOR can be potently inhibited by the fungal metabolite rapamycin, which acts on mTORC1 upon binding to an endogenous protein, FKBP12 [93-95]. Long-lasting treatments with rapamycin have been reported to inhibit mTORC2 in a relatively mild and cell type-dependent manner [103].

The existence of this potent inhibitor has facilitated the study of the mTORC1 complex, which is far better characterized than mTORC2. mTORC1 is involved in the regulation of cell growth and proliferation, ribosome biogenesis and translation of a subset of mRNAs, cellular energy responses and autophagy [93-95]. mTORC2 was initially described as a regulator of the cellular actin cytoskeleton [99], and the relatively recent discovery that mTORC2 is the kinase responsible for regulating the phosphorylation of serine 473 of Akt/PKB has highlighted its major role in the regulation of apoptosis [96,104].

### mTORC1

mTORC1 is activated by the small GTPase Rheb when it is in a GTP-bound state (Figure 2A) [95]. This activation mechanism is tightly controlled by the GAP activity of tuberin, the gene product of TSC2 (see below) [95]. Tuberin must be associated to hamartin, the TSC1 gene product (see below), to achieve full activation; this complex favors a GTP-to-GDP conversion of Rheb, which results in the inactivation of Rheb and the inhibition of mTORC1. A variety of signaling pathways act on the mTORC1 cascade by regulating the assembly or the activity of the tuberin/hamartin complex (Figure 2A). Thus, several signals converge to act on the TSC1/TSC2 complex and phosphorylate either tuberin or hamartin to regulate their activity [95]. Some of these phosphorylation events enhance the activity of tuberin and hamartin, leading to downregulation of the mTORC1 pathway, whereas other events inhibit their activity, resulting in upregulation of the mTORC1 cascade (Figure 2A) [95]. Several of the kinases capable of inducing the activation of mTORC1 through the phosphorylation of tuberin are regulated by tyrosine kinase receptors (RTK). In particular, Akt, RSK1 and the ERKs can all phosphorylate different residues of tuberin, leading to the inactivation of the tuberin/hamartin complex activity towards Rheb [95]. In addition, GSK3β, AMPK and REDD1 are among the pathways capable of inhibiting the activation of mTORC1 by acting on tuberin [95]. In general, the availability of rich energy sources and growth-favouring conditions converge to activate the mTOR cascade, whereas critically minimal energy conditions shut it off. In addition, mTORC1 functions as a sensor for amino acids; it is activated in the presence of amino acids, but inhibited in their absence, and this effect is mediated by the small GTPase Rag (Figure 2A) [105].

### mTORC2

A second mTOR-containing complex (mTOR Complex 2, mTORC2) was defined upon identification of a new mTOR interactor, Rictor [99]. Rictor binds to a pool of mTOR protein that is distinct from that bound by Raptor: these two adaptors define the two mTOR-containing complexes (Figure 2A) [97,99]. It has been demonstrated that mTORC2 regulates PKCα and the actin cytoskeleton [99]. The major break-through in the study of mTORC2 function came from the identification of this complex as the kinase capable of phosphorylating the hydrophobic motif of Akt/PKB (serine 473 in Akt1), a serine/threonine kinase whose activation had long been known to be phosphatidylinositol-3-kinase-dependent under physiological conditions [104]. In the accepted model of Akt activation, the PH domains of Akt and phosphatidylinositol-dependent kinase 1 (PDK1) would both bind PIP3 domains upon the activation of PI-3-kinase in cells and the generation of phosphatidylinositol-3,4,5-phosphate (PIP3) at the plasma membrane, thus bringing Akt and PDK1 into close proximity and favouring the phosphorylation of threonine 308 in Akt by PDK1 [106]. In order to reach full activation, Akt must be phosphorylated at both serine 473 and threonine 308 [106]. Although the identity of the kinase responsible for phosphorylating the former had been elusive for a long time, this enzyme was known to be PI-3-kinase-dependent and was termed 'PDK2'. The discovery that mTORC2 is this kinase (or the most prominent one) both *in vitro *[104] and *in vivo *[107,108] has opened important retrospective interpretations of the literature. Although the basis for the sensitivity of mTORC2 to PI-3-kinase still remains poorly understood, the sensitivity might not be dependent on the relocalization of the molecules involved because treatment with PI-3-kinase inhibitors is sufficient to block *in vitro *phosphorylation of Akt at serine 473 by the isolated mTORC2 complex [104].

Finally, emerging evidence suggests that the serine kinase SGK1 (serum- and glucocorticoid-induced kinase 1), which belongs to the same family of kinases as Akt, can be phosphorylated by mTOR, most likely when associated with mTORC2 [96,109].

### Cross-regulation of mTORC1 and mTORC2

Besides containing the same core kinase component (mTOR), the two complexes regulate one another. Several studies have shown that at least three distinct tuberin residues can be phosphorylated by Akt [95]. These phosphorylation events inhibit tuberin activity and its association with hamartin, leading to enhanced GTP-Rheb activity and increased mTORC1 activity. This is a robust and common mechanism of mTORC1 activation by tyrosine kinase receptors [95]. Given that Akt itself is phosphorylated by mTORC2, as discussed above, one might imagine that mTORC1 could be activated by mTORC2 (Figure 2A). However, studies *in vivo *in Rictor-mutant mice have shown that phosphorylation of Akt at Ser473 is almost completely abrogated in the absence of mTORC2, whereas phosphorylation at Thr308 remains normal [107,108]. In these mice, phosphorylation of tuberin at Akt-specific sites was not altered [107]. In addition, the phosphorylation levels of S6K at Thr389 (a readout of mTORC1) were only minimally affected [108]. These data have revealed the possibility that Akt could be activated simply by phosphorylation at Thr308 and that this event is sufficient to determine its activity towards some of its substrates, including tuberin [108]. In this case, mTORC2 cannot be positioned upstream of mTORC1 in a simple, linear manner.

A very strong cross-talk mechanism has been identified due to the observation that upregulation of mTORC1 activity in tumours derived from TSC patients and in cells lacking *Tsc2 *is accompanied by downregulation of Akt [110-114]. A novel negative feedback loop has been described that is activated by mTORC1 and is believed to be protective of TSC tumours. It was shown that S6K1 activity leads to inhibition of both PDGF and insulin signaling [110-113]. In the case of PDGF it was shown that in the absence of the *Tsc2 *gene, increased S6K1 activity leads to downregulation of the PDGF receptor levels [110]. In the case of the insulin response, it was demonstrated that S6K1 phosphorylates the adaptor molecule insulin receptor substrate (IRS), a key mediator of the insulin receptor response, causing its degradation and a downregulation in signaling towards both Akt and the ERKs (Figure 2A) [110-113]. A recent study has suggested that the last feedback loop might be stronger than initially appreciated. A novel molecule, DEPTOR, has been identified and shown to associate with and to inhibit both mTORC1 and mTORC2 when isolated and assayed by *in vitro *kinase assays [96]. However, when overexpressed in cells or in naturally occurring multiple myelomas, DEPTOR potently inhibits mTORC1, resulting in upregulation of mTORC2/Akt via the S6K-mediated feedback loop [96]. It has been proposed that this mechanism is so strong in cells and tissues that it overrides the inhibitory activity of DEPTOR on mTORC2 [96]. Therefore, through a negative feedback loop, mTORC1 is able to potently inhibit mTORC2 (Figure 2A). More recently, S6K1 was found to directly phosphorylate rictor, possibly resulting in downregulation of the mTORC2 activity towards Akt Ser473 through a mechanism that does not appear to directly influence mTORC2 kinase activity *in vitro *[114]. The precise molecular mechanisms of this cross-regulation remain to be elucidated, but these studies provide evidence of an additional negative feedback loop that allows for cross-talk between mTORC1 and mTORC2 [114].

The existence of these negative feedback loops might also have important implications for therapy. Treatment with rapamycin causes upregulation of mTORC2 and Akt [111]. Although prolonged exposure to rapamycin might counteract this upregulation by inhibiting mTORC2, this effect appears to be cell type-dependent [103]. Therefore, the possible drawbacks of using rapamycin should be taken into serious consideration when designing therapeutic interventions.

## Dysregulation of mTORC1 in polycystic kidney disease

Several studies have suggested that the mTORC1 cascade might be dysregulated in polycystic kidney disease. Three independent groups have shown that rapamycin has beneficial effects and diminishes the cystic index in rodent models of polycystic kidney diseases [26,29,30]. It should be noted that none of the animal models that were shown to be sensitive to rapamycin develop polycystic kidney disease due to mutations in either *Pkd1 *or *Pkd2*.

However, retrospective analysis of ADPKD patients, who underwent renal transplantation and were receiving rapamycin derivatives as an immuno-suppressive therapy, revealed a significant reduction in the renal volume of the polycystic kidneys that had not been removed [26]. In addition, the epithelium of both ADPKD cystic kidneys and *Pkd1 *mutant kidneys displayed enhanced mTORC1 activity, as evidenced by immunohistochemical analysis of S6K and mTOR phosphorylation levels [26]. These initial studies prompted several centres to design pilot clinical studies to determine the efficacy of rapamycin treatment on ADPKD patients.

Subsequent studies have further confirmed that mTORC1 effectors, such as S6Rp, are strongly phosphorylated in the epithelium lining the cysts of ADPKD tissues [27] and in animal models [28]. However, mTORC1 upregulation was not observed in all of the cysts, but only in a subset of the cystic epithelia, in both humans [27] and mice [[28] and M Pema and A Boletta, unpublished]. These data highlight the fact that the interconnection between cyst formation and dysregulation of the mTORC1 cascade is more complicated than initially anticipated. Moreover, based on these data, treatment with rapamycin is expected to be effective only on a subset of cysts.

A more recent report has shown that inactivation of the *Tsc1 *gene in the kidney results in massive renal cystogenesis [115]. In this case, as expected, all of the cells in which the *Tsc1 *gene was inactivated displayed enhanced mTORC1 activity [115]. However, wild type epithelial cells that did not show Cre recombinase activity (and, therefore, loss of the *Tsc1 *gene) and, consequently, did not show upregulation of mTORC1 can be found in the cysts, suggesting a chimeric formation of renal cysts. In the same report, inactivation of the *PTEN *gene resulted in only minimal upregulation of the mTORC1 cascade, which was insufficient to cause renal cystogenesis. Based on their data, the authors proposed that cyst formation requires upregulation of the mTORC1 cascade, but that the PI3k/Akt pathway does not appear to be the major regulator of mTORC1 in the kidney [115].

However, the fact that several cysts in ADPKD tissue and *Pkd1 *mutant kidneys appear to be negative to upregulation of mTORC1 calls into question the essential role of this cascade in the formation of renal cysts. This finding suggests that mTORC1 upregulation is unlikely to be an initiating event of cystogenesis, even though it might contribute to cyst growth and expansion, which could explain the beneficial effects of rapamycin.

Additional studies are necessary in order to further investigate the relationship between the mTORC1 cascade and renal cyst formation and expansion.

## Regulation of the mTOR complexes by the polycystins

In addition to showing that mTORC1 is upregulated in the cystic epithelia of ADPKD tissues and in rodent models of PKD, a recent study has demonstrated that the C-terminal tail of PC-1 interacts with tuberin [26]. Based on these findings, it has been hypothesized that PC-1 might regulate the mTORC1 pathway [26].

Direct experimental evidence that PC-1 is able to inhibit the mTORC1 cascade has recently been provided by our group [87]. Overexpression of full-length PC-1 in renal epithelial cells (MDCK type II) and in fibroblasts was shown to reduce cell size by inhibiting mTORC1 and its two targets, S6K1 and 4EBP1 (Figure 2B) [87]. The opposite effect was found in several sets of fibroblasts lacking the *Pkd1 *gene, isolated from two different mouse models [87]. PC-1 was also shown to inhibit the mTORC1 cascade in a tuberin-dependent manner by regulating its ERK-dependent phosphorylation [87]. Based on these studies using gain- and loss-of-function cellular systems, it was proposed that PC-1 regulates the mTORC1 cascade by signaling to tuberin primarily via the ERK cascade, although additional signaling pathways might contribute as well [87]. These data show that PC-1 is able to inhibit the mTORC1 cascade in renal epithelial cells and fibroblasts (Figure 2B). It should be noted that different results have been reported by a different group [27]. Using a single set of *Pkd1*^+/+ ^and *Pkd1*^-/- ^MEFs immortalized by knocking-down *p53*, Hartman *et al*. reported that absence of *Pkd1 *gene expression under these conditions did not result in defective mTORC1 signaling in fibroblasts [27]. However, enhanced mTORC1 signalling was observed in the epithelia lining the cysts of ADPKD specimens [27]. Based on these findings, PC-1 was proposed to regulate the mTOR cascade in a cell-type dependent manner [27]. Further studies are required to reconcile these discrepancies.

In addition to the role of PC-1 in the regulation of the mTORC1 complex, several lines of evidence suggest that the regulation of mTOR by PC-1 is not limited to mTORC1.

In previous studies, PC-1 was shown to induce phosphorylation of Akt at both Thr308 and Ser473; Akt, in turn, phosphorylates its target FKHR (FOXO1) to achieve resistance to apoptosis [81]. In addition, PC-1 was proven to cause rearrangements of the actin cytoskeleton in order to control cell migration via PI3K/Akt [70]. Furthermore, in agreement with these results, Yamaguchi *et al*. have shown reduced phosphorylation of Akt at Ser473 in M-1 cells and normal human kidneys cells following calcium restriction [116] as well as in the cyst-lining epithelium isolated from ADPKD tissues [116].

Taken together, these data strongly suggest that PC-1 induces the activation of mTORC2 (which is responsible for phosphorylation of Akt Ser473) and that the activity of this complex might be impaired in the absence of *PKD *gene expression, at least in cystic kidneys.

In contrast, the epithelia lining the cysts in ADPKD liver tissue display enhanced phosphorylation of Akt at Ser473 [31]. One possible explanation for this finding is that Akt and its activity towards the mTOR cascade might be regulated differently in different tissues. Although this is certainly a possibility, it should also be taken into account that the levels of Akt activation in these liver cysts have only been evaluated by immunohistochemistry [31]; biochemical means would probably be more accurate and more sensitive.

Therefore, retrospective examination of past work in light of recent discoveries in mTORC1 and mTORC2 complex biology leads to the conclusion that PC-1 inhibits mTORC1, but might activate mTORC2 (Figure 2B), perhaps in a cell-type dependent manner. However, formal evidence that this hypothesis is correct is still lacking. Future studies should focus on examining the activity of the two mTOR complexes in gain- and loss-of-function cellular systems and in cystic tissues. In addition, the role of the two complexes in PC-1 function should be investigated by manipulating the key components of mTORC1 (Raptor) [97] and mTORC2 (Rictor) [99].

One important question that arises from all of the above studies is how PC-1 could differentially regulate the mTORC1 and mTORC2 complexes. Interesting insights might be derived from the function of the TSC proteins. In fact, although the hamartin/tuberin complex is a potent inhibitor of the mTORC1 complex, it can regulate the activation state of mTORC2 on at least two different levels: (i) it directly binds to and stimulates the activity of mTORC2 [117]; and (ii) it can act on mTORC2 activity indirectly, via the S6K-mediated feedback loop [111]. As stated above, the latter is a rather strong signal capable of potently regulating the expression levels of the PDGF receptor [110] and the insulin response by controlling the degradation rate of the adaptor IRS [113]. Therefore, cells lacking *Tsc2 *display strong upregulation of mTORC1 and downregulation of mTORC2. This effect is due to both hyperactivation of the S6K-mediated feedback loop [111] and absence of the tuberin/hamartin complex associated with mTORC2 [117].

In a similar manner, PC-1 inhibits mTORC1, which might release the S6K1 feedback loop, resulting in the activation of mTORC2 and Akt. A second possibility is that PC-1 regulates tuberin activity towards both mTOR complexes.

Finally, the role of PC-2 in regulation of mTOR by PC-1, if any, remains to be investigated. Because the two proteins have been proposed to perform most of their functions in a complex, it is highly likely that PC-2 will play a key role in the regulation of this cascade as well. Future studies should focus on this aspect.

## Cross-talk between the PKD and TSC genes and proteins

The two major regulators of the mTORC1 cascade, *TSC1 *(on chromosome 9) and *TSC2 *(on chromosome 16), are the genes mutated in a genetic disease called Tuberous Sclerosis Complex (TSC) [118]. This disease is characterized by the formation of hamartomas (benign tumour lesions) in several organs, including the brain, skin, heart, lung and kidney. TSC is inherited in an autosomal dominant manner, but a second hit affecting the normally inherited allele or loss of heterozygosity (LOH) has been described in some of the lesions [118].

TSC can present with a severe renal phenotype, including angiomyolipomas and sporadic bilateral renal cyst formation [119]. In a few cases, severe polycystic kidney disease can be observed. Of interest, the *PKD1 *and *TSC2 *genes are located very close to each other on the human chromosome 16 in a tail-to-tail orientation [120]. Furthermore, this genomic structure is conserved in mice. Molecular analysis has revealed that, in the majority of cases of severe polycystic kidney disease observed in TSC, large deletions of chromosome 16 affecting both the *TSC2 *and the *PKD1 *gene can be observed [120]. Thus, a novel syndrome, called TSC/PKD contiguous genes syndrome, has been defined as a separate entity [120]. It is important to note that the PKD phenotype in these patients appears to be very severe [119,120], with massive enlargement of the kidneys during childhood, suggesting that deletion of both *TSC2 *and *PKD1 *genes has an additive effect in the kidney. In addition, the aforementioned studies performed in *Pkd1 *and *Tsc2 *double heterozygote mice [28] further reinforce the idea of a possible cross-talk between the *PKD *and *TSC *genes.

The first indications that the *PKD *and *TSC *genes are, indeed, functionally linked came from studies by the group of Dr C Walker on the Eker rat model [121]. This rat model carries mutations in the *Tsc2 *gene, mimicking many of the features of TSC, and develops a severe renal cystic phenotype. It was found that renal epithelial cells derived from the cysts display inactivation of both *Tsc2 *alleles [121]. Using an antibody directed against endogenous PC-1, it was found that PC-1 is retained in the Golgi compartment in these cells. Replacing wild-type tuberin completely rescued trafficking of PC-1 to cell-cell junctions [121].

**Figure 3:**
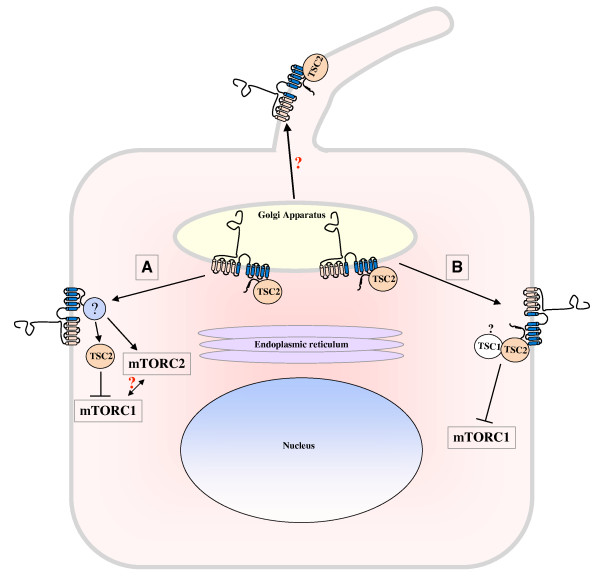
**Functional cross-talk between the *TSC2 *gene product, Tuberin and the *PKD1 *gene product, polycystin-1**. Evidence published to date suggests that PC-1 trafficking from the Golgi compartment to cell-cell junctions requires Tuberin [122]. The role of Tuberin in PC-1 trafficking to the primary cilium was not investigated. In addition, PC-1 can regulate the mTORC1 cascade by regulating the phosphorylation and activity of Tuberin (**A**) [87]. Furthermore, a physical interaction was described between the portion of the PC-1 cytoplasmic C-terminal tail most proximal to the last transmembrane domain and Tuberin [26]. The PC-1/Tuberin interaction might be necessary for the correct trafficking of PC-1 or for regulation of mTORC1 (**B**, see text). Future studies should focus on providing experimental evidence of the significance of this interaction. One final possibility is that PC-1 and Tuberin cross-talk at several levels.

These data suggest that PC-1 and tuberin are able to functionally cross-talk (Figure 3) and that PC-1 might act downstream of tuberin. According to this model, renal cyst formation in TSC might be due to defective PC-1 activity.

More recently, Shillingford *et al*. have shown that a short C-tail of PC-1 expressed as a chimera with the CD16 signal peptide and the CD7 transmembrane domain co-localizes with mTOR and tuberin in the Golgi compartment [26]. In addition, a portion of the intracellular C-tail of PC-1 co-immunoprecipitates with tuberin [26]. Evidence that the full-length, endogenous PC-1 interacts with tuberin has not been reported to date. Nevertheless these data, along with the enhanced activity of mTORC1 observed in cystic epithelia, prompted the authors to suggest that PC-1 and tuberin form a complex to regulate mTORC1 [26]. In the proposed model, PC-1 is a constitutive partner of the tuberin/hamartin complex that is required for its GTPase activity towards Rheb. In the absence of PC-1, the tuberin/hamartin complex would not be functional, leading to enhanced mTORC1 activity (Figure 3B).

In a more recent study, it was shown that PC-1 regulates the mTORC1 pathway and that it requires tuberin to do so [87]. PC-1 induces downregulation of the ERKs through a yet-to-be-identified mechanism. This inhibition results in the decreased phosphorylation of tuberin at ERK-dependent sites, which leads to enhanced GTPase activity towards Rheb and the inhibition of mTORC1 [87]. According to this model, tuberin acts downstream of PC-1, at least in its activity towards mTORC1 (Figure 3A).

Can we place these results into a single framework in order to better understand the reciprocal regulation of the two genes? The idea of an interaction between PC-1 and tuberin that is able to regulate PC-1 trafficking is very appealing because it would easily explain the renal phenotype observed in TSC/PKD contiguous genes syndrome [121]. If PC-1 requires tuberin to localize to the plasma membrane, where it carries out its key functions, then we can imagine that double heterozygosity might be sufficient to cause cyst formation. In this scenario, the cells express a half amount of PC-1, only part of which is correctly delivered to the plasma membrane, thus causing a drop in PC-1 function/signaling below the critical threshold of activity necessary to prevent cyst formation. If this were the case, one would expect that cells carrying heterozygous inactivation of the *TSC2 *gene should exhibit defective PC-1 trafficking or function. This hypothesis needs to be formally tested, although analysis of the distribution of endogenous PC-1 poses several challenges due to the limited reliability of the available antibodies directed against this protein.

One additional caveat of this model is that the expression level of the tuberin/hamartin complex appears to be much higher than that of PC-1, and a halved expression of tuberin should be more than sufficient to deliver PC-1 to the plasma membrane. If tuberin interacts with PC-1, however, we cannot assume that the stoichiometry of the complex is 1:1. Recent studies have revealed a 1:3 stoichiometry for the PC-1:PC-2 complex [40]. Therefore, although PC-2 appears to be expressed at higher levels than PC-1, this difference might be biologically justified by the composition of the complex. Similar considerations might apply to the PC-1/tuberin interaction in support of the proposed model.

Finally, one possibility to be considered is that the TSC/PKD cross-talk acts at several levels. On one hand, tuberin might mediate an essential trafficking step of PC-1. On the other hand, PC-1 at the plasma membrane might control the mTORC1 cascade through regulation of tuberin, either via physical association with this protein or through indirect regulation of its activity (Figure 3).

Although these studies have begun to shed light on this complicated inter-relationship, it is clear that intense efforts will be required to gain further insight into PKD/TSC gene cross-talk and its biological effects. In particular, efforts should be devoted to clarify if over-expressed and/or endogenous, full-length PC-1 and tuberin are indeed able to associate in a complex in cells and tissues. In addition, it will be important to understand if PC-1/tuberin cross-talk is limited to mTORC1 regulation or if it is involved in additional biological functions. In fact, the recent finding that spontaneous renal cystogenesis is enhanced in double Tsc2^+/-^;Pkd1^+/- ^mice, but that some of the cysts observed are negative for mTORC1 upregulation strongly suggests that the cross-talk extends beyond regulation of the mTORC1 cascade [28].

## Cilia, the cell cycle and cell size

As stated above, the PC-1/2 complex localizes to cilia in different mammalian cell types [72,73]. Therefore, it is expected that at least some of its functions will coincide with those of the cilia. The structure of cilia and flagella, as well as that of the key components of the intraflagellar transport (IFT) machinery, is highly conserved in all eukaryotes. For this reason, the unicellular alga *Chlamidomonas reinhardtii *has been used extensively to gain important insights into the function of cilia in higher eukaryotes.

Previous studies performed in this organism have demonstrated that there is a strong correlation between the length of flagella, the size of cells and the ability of cells to undergo division [122]. Based on these findings, it was proposed and subsequently demonstrated that the cilium plays a fundamental role in the regulation of cell cycle progression [122,123], both in *Chlamidomonas *[122,123] and in mammalian cells [124]. The studies showing that PC-1 is able to decrease cell size via the mTORC1 cascade, in addition to its ability to slow progression through the cell cycle [87], parallel similar studies performed by the group of Dr Quarmby. This group has shown that the orthologs of NIMA (never in mitosis) kinases in *Chlamidomonas *achieve a very similar effect [123]. The regulation of cell size in lower eukaryotes is much easier to study and understand than in higher eukaryotes; indeed, our understanding of how cell and tissue size are established in higher eukaryotes and mammals is far less intuitive. Nevertheless, there might be interesting parallels worth following, including the correlation between cell size, the cell cycle and flagellar size. Intriguingly, hamartin has recently been localized to the basal body of cilia in mammalian cells [27], and absence of the hamartin/tuberin complex has been linked to a long ciliary phenotype [27]. This effect was rapamycin-insensitive, suggesting that mTORC1 does not regulate cilia length [27]. These studies, however, do not exclude the possibility that cilia might act upstream of mTORC1 and regulate its activity.

Is there a link between cilia/flagella length, cell size, cell division and TORC1 activity? It is tempting to speculate that perhaps cilia are sensory organelles capable of sensing their environment to coordinate protein synthesis and cell division in order to maintain proper cell size. This effect might be achieved through the TORC1 cascade, which is known to regulate cell size in virtually all systems in which it has been studied [93-95]. *Chlamidomonas *would be the ideal system in which to test this hypothesis because TORC1 is expressed, functional and sensitive to rapamycin in this organism, although it has been only minimally studied [125].

## Conclusion

Recent studies have uncovered important roles for Polycystin-1 in the regulation of the mTOR cascades and its complexes. Not only have these studies shown the potential of using a well-characterized drug, rapamycin, to slow disease progression in ADPKD, but they have also uncovered important new functions of the polycystins.

One exciting aspect of these recent studies is that they have revealed an important cross-talk between the genes mutated in ADPKD and those mutated in TSC, although further studies are required to fully understand the molecular details of this relationship. It is important to note that although dysregulated growth and proliferation (possibly driven by mTOR in some cysts) might be important components of renal cyst expansion in human patients and potentially good targets to slow disease progression, they might not be the initiating events of cystogenesis. Increasing evidence suggests that defective planar cell polarity (PCP) might cause cyst formation [33-37]. The elucidation of the molecular mechanisms involved in this process, which are defective in cystic kidney diseases, might allow for the identification of potential pharmacological targets that would then enable the design of a specific cure for the disease.

Finally, one intriguing aspect discussed in this review is the speculative link between cilia, cell size and regulation of the cell cycle. What is the relationship between the regulation of cell size and the pathogenesis of TSC and/or ADPKD? There is currently no answer to this question, but it cannot be ignored that besides the TORC1 pathway, the other known cascade involved in the regulation of cell/tissue size is that of the *Hippo *pathway [126]. This pathway is the main cascade lying downstream of the Fat cadherins, master regulators of PCP, as stated above [127]. There is a well-documented link between programs of cell polarity and programmes of cell growth [128]. It is intuitive that these pathways need to be coordinated in order to achieve and maintain proper tissue morphogenesis. This link might be regulated by the primary cilium and could be disrupted in cystic kidney diseases or other disorders such as cancer.

## Abbreviations

ADPKD: autosomal dominant polycystic kidney disease; AMPK: AMP-dependent protein kinase; AP-1: activator protein 1; DEPTOR: DEP domains interactor of mTOR; eIF4: eukaryotic translation initiation factor 4; eIF2: eukaryotic translation initiation factor 2; ER: endoplasmic reticulum; ERK: extracellular regulated kinase; FKBP12: FK506-binding protein 12; FKHR: forkhead rabdomyosarcoma; FOXO: Forkhead box class O; GAP: GTPase-activating protein; GEF: guanine-nucleotide exchange factor; GPS: G-protein coupled proteolytic site; GSK3β: glycogene synthase kinase β; IFT: intra-flagellar transport; IRS: insulin receptor substrate; JAK: Janus kinase; LST8: lethal with SEC13 protein 8; MDCK: Madin Darby kidney cells; mTOR: mammalian target of rapamycin; NFAT: nuclear factor of activated T-cells; NIMA: never in mitosis; PCP: planar cell polarity; PDK1: phosphatidylinositol-dependent kinase 1; PERK: PKR-like ER kinase; PH domain: plecstrin homology domain; PI3K: phosphoinositide 3-kinase; PKCα: protein kinase Cα; PRAS40: proline rich Akt substrate; Protor: protein observed with rictor-1; Rag: Ras related GTPase; Raptor: regulatory associated protein of mTOR; REDD1: DNA damage response 1; Rheb: Ras homologue enriched in brain; Rictor: rapamycin-insensitive companion of mTOR; RSK: p90 ribosomal protein S6 kinase; SGK1: serum- and glucocorticoid-induced kinase 1; STAT: signal transducers and activators of transcription; S6K: 70 kDa ribosomal protein S6 kinase; TRPL: transient receptor potential; TSC: tuberous sclerosis complex; 4E-BP1: eukaryotic-translation-initiation-factor-4E-binding protein 1.

## Competing interests

The author declares no competing interests.

## Authors' contributions

AB wrote the manuscript and prepared the figures.
